# Accramycin A, A New Aromatic Polyketide, from the Soil Bacterium, *Streptomyces sp.* MA37

**DOI:** 10.3390/molecules24183384

**Published:** 2019-09-17

**Authors:** Fleurdeliz Maglangit, Qing Fang, Valentin Leman, Sylvia Soldatou, Rainer Ebel, Kwaku Kyeremeh, Hai Deng

**Affiliations:** 1 Marine Biodiscovery Centre, Department of Chemistry, University of Aberdeen, Meston Walk, Aberdeen AB24 3UE, Scotland, UK; r01qf16@abdn.ac.uk (Q.F.); sylvia.soldatou@abdn.ac.uk (S.S.);; 2 College of Science, University of the Philippines Cebu, Lahug, Cebu City 6000, Philippines; 3 Organic Chemistry Division, SIGMA Clermont, 27, Rue Roche Genes, 63170 Aubiere, France; valentin.leman@sigma-clermont.fr; 4 Department of Chemistry, University of Ghana, P.O. Box LG56 Legon-Accra, Ghana; kkyeremeh@ug.edu.gh

**Keywords:** accramycin, naphthacene, type II polyketide, *Streptomyces sp.* MA37, Group B *Streptococcus*

## Abstract

Drug-like molecules are known to contain many different building blocks with great potential as pharmacophores for drug discovery. The continued search for unique scaffolds in our laboratory led to the isolation of a novel Ghanaian soil bacterium, *Streptomyces sp*. MA37. This strain produces many bioactive molecules, most of which belong to carbazoles, pyrrolizidines, and fluorinated metabolites. Further probing of the metabolites of MA37 has led to the discovery of a new naphthacene-type aromatic natural product, which we have named accramycin A **1**. This molecule was isolated using an HPLC-photodiode array (PDA) guided isolation process and MS/MS molecular networking. The structure of **1** was characterized by detailed analysis of LC-MS, UV, 1D, and 2D NMR data. Preliminary studies on the antibacterial properties of **1** using Group B *Streptococcus* (GBS) produced a minimum inhibitory concentration (MIC) of 27 µg/mL. This represents the first report of such bioactivity amongst the naphthacene-type aromatic polyketides, and also suggests the possibility for the further development of potent molecules against GBS based on the accramycin scaffold. A putative *acc* biosynthetic pathway for accramycin, featuring a tridecaketide-specific type II polyketide synthase, was proposed.

## 1. Introduction

Natural products continue to provide highly diverse chemical scaffolds, which have facilitated the research and development of many currently marketed drugs and viable clinical candidates [1]. Excellent examples of these natural scaffolds that have aided in the design of several compound libraries include the β-lactams (penicillin G), macrolactones (erythromycin), glycopeptides (vancomycin), tetracylines, and modified nucleosides [1,2]. 

Bacterial aromatic polyketides are prolific sources of many diverse scaffolds, some of which constitute a major class of antibiotics known to be generated on enzymatic assembly lines. For example, fasamycins and naphthacemycins contain 1-phenylnaphthacene moiety, which is known to have a rare carbon skeleton, and is a potent scaffold [3]. The *gem*-dimethyl tetracyclic carbon framework in these compounds has been reported to show potent anti-methicillin-resistant *Staphylococcus aureus* (MRSA) activities. Furthermore, fasamycins A and B were found to inhibit the FabF enzyme of type II fatty acid (FASII) biosynthesis in bacteria [4], while naphthacemycins showed inhibition against several MRSA and methicillin-susceptible *Staphylococcus aureus* (MSSA) strains, in addition to an efficient circumvention of β-lactam resistance when administered in combination with imipenem [5,6,7]. As part of our ongoing screening program to search for unique scaffolds, we have identified a cluster of metabolites containing the rare phenylnaphthacene carbon framework from the soil bacterium, *Streptomyces sp.* MA37, which is a prolific producer of natural products including legonmycins [8], neocarazostatin A [9,10,11], legonoxamines [12], and a variety of organofluorines [13,14]. 

The current study establishes MS/MS molecular networks (MN) coupled with HPLC-UV-based techniques for the rapid chemical dereplication and structural feature identification of metabolites from complex crude microbial extracts of *Streptomyces sp*. MA37. The streamlined MN dereplication process was performed via the online Global Natural Product Social (GNPS) Molecular Networking platform [15,16]. Compounds with structurally-related motifs are clustered together in the network based on a similarity in the mass spectra, including fragmentation patterns, and when coupled with HPLC-UV, this provided an easy and rapid tool for the effective isolation of target scaffolds. This technique is very important, since dereplication and subsequent identifications of known motifs at an early stage in natural product research is a crucial step for the accelerated isolation and characterization of new chemical scaffolds, thereby saving enormous amounts of time, effort, and cost [17,18]. Subsequently, in the MA37 molecular network, a cluster of compounds was identified that showed characteristic UV patterns (226, 250, 286, 355, and 420 nm), suggesting a common chromophore [3,4,19]. Manual dereplication using AntiBase (2012) indicated that members of this cluster have not been previously reported. 

The isolation was carried out and afforded accramycin A **1**. This molecule was named as such due to the origin of the producer strain *Streptomyces sp.* MA37 from the Legon–Accra region in Ghana. Using LC-MS, UV, 1D, and 2D NMR techniques, the structure of **1** was determined to be an aromatic naphthacene-based natural product. When tested against Group B *Streptococcus*, accramycin A **1** showed a minimum inhibitory concentration (MIC) of 27 ug/mL. To our knowledge, this activity has never been reported in any naphthacene-type congeners, suggesting that the accramycin scaffold could be used as a basis for the development of potent drugs against Group B *Streptococcus* (GBS). Two further accramycin analogues, **2** and **3** (Figures S10–S14) were also identified in fraction S3; however, they were not isolable. Our results underline the enormous potential of the MS/MS molecular network coupled with UV-based approaches to identify and target unprecedented scaffolds.

## 2. Results and Discussion

**Figure 1:**
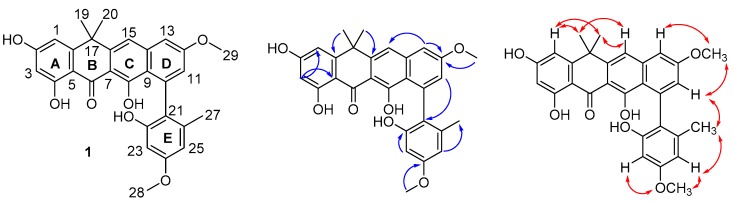
Structure of accramycin A (**1**) with key HMBC (→) and NOESY (↔) correlations in DMSO-*d_6_*_._

A large fermentation (12 L) of *Streptomyces* sp. MA37 was performed at 28 °C at 180 rpm for 7 days. Subsequently, Diaion® HP-20 (3 g/50 mL) was added to the fermentation culture and left to shake overnight under the same incubation conditions. The mixture was filtered under vacuum, after which the resin and mycelia were extracted with 100% methanol. The methanol extract was concentrated under vacuum to give a total crude extract, followed by fractionation using Strata^®^ C18-E solid phase extraction (SPE), which yielded six fractions (S1–6). All the fractions obtained were subjected to HPLC analysis with diode array detection to screen for metabolites bearing unique scaffolds. Fraction S3 showed the characteristic naphthacene UV spectra (226, 250, 286, 355, and 420 nm); hence, it was selected for further fractionation using semi-preparative reversed-phase HPLC, resulting in the isolation of accramycin A **1** (2.0 mg) (Figure 1).

### 2.1. Structure Elucidation

Accramycin A **1** was obtained as a yellow powder. It showed UV absorption maxima at 226, 250, 286, 355, and 420 nm, which is distinctive of a fasamycin or naphthacemycin congener [5,19]. The molecular formula of **1** (Figure 1) was established as C_29_H_26_O_7_ by HR ESIMS (calculated [M + H]^+^ = 487.1751; observed [M + H]^+^ = 487.1741; ∆ = −2.1 ppm), indicating 17 degrees of unsaturation (Figures S1 and S2). 

^1^H and ^13^C-NMR spectra and HSQC analysis revealed the presence of 29 carbons, including three methyl carbons at *δ*_C_ 20.6, 34.4, and 34.7; two methoxy signals at *δ*_C_ 55.3 and 55.6; seven sp^2^ methines at *δ*_C_ 99.2, 104.0, 106.6, 106.9, 112.1, 115.8, and 121.7; one sp^3^ quaternary carbon at *δ*_C_ 39.3; and sixteen sp^2^ quaternary carbons (*δ*_C_ 105.0–189.2) (Table 1, Figures S3–S9). Detailed analysis of the spectral data suggested that **1** was closely related to the previously described naphthacene, fasamycin C [19]. 

The long-range couplings observed in the HMBC spectrum established the connectivity of rings A–D. The cross-peaks from H-1 (*δ*_H_ 6.49) to C-3 (*δ*_C_ 104.0) and C-5 (*δ*_C_ 105.0) revealed a 2,4-dihydroxy-substituted benzene substructure (ring A). The cross-peaks from H_3_-19 (*δ*_H_ 1.70) and H_3_-20 (*δ*_H_ 1.69) to C-16 (*δ*_C_ 147.4), C-17 (*δ*_C_ 39.3), and C-18 (*δ*_C_ 154.5) indicated a dimethyl motif attached to carbon 17 (ring B), and connected to ring A via C-5 and C-18. Carbon 6 resonated at δ_C_ 189.2, which is consistent with a ketone moiety conjugated to two aromatic rings in the structure. The cross-peaks from H-15 (*δ*_H_ 7.47) to C-7 (*δ*_C_ 107.4), C-9 (*δ*_C_ 118.7), and C-17 (*δ*_C_ 39.3) established the nature of ring C, which was condensed to ring B via C-7 and C-16. H-11 (*δ*_H_ 6.71) and H-13 (*δ*_H_ 7.19) in ring D were found to be arranged *meta* to one another based on their coupling constant (2.4 Hz). The HMBC correlations from H_3_-29 (*δ*_H_ 3.95) to C-12 (*δ*_C_ 161.1), and H_3_-28 (*δ*_H_ 3.80) to C-24 (*δ*_C_ 160.2) located the two methoxyl functions in rings D and E, respectively. Furthermore, ring E displayed two *meta*-coupled protons, H-23 (δ_H_ 6.32) and H-25 (δ_H_ 6.37, (*J* = 2.4 Hz), and an aromatic methyl group (*δ*_H_ 1.91, H_3_-27). The arrangement of the different substituents within this aromatic ring was confirmed by HMBC correlations from H_3_-27 to C-21 (*δ*_C_ 125.3), C-25 (*δ*_C_ 106.9), and C-26 (*δ*_C_ 138.2), and H-23 to C-21, C-22 (*δ*_C_ 155.2), and C-24 (*δ*_C_ 160.2), while the attachment of ring E to the rest of the molecule was established by the diagnostic correlation between H-11 and C-21.

The structure of **1** was further corroborated by analysis of the NOESY spectrum acquired in DMSO-*d_6_* (Figure 1) and by comparison with the MS and NMR data of fasamycins and naphthacemycins in the literature [5,19], from which **1** differs with respect to the presence of a methoxy moiety (δ_H_ 3.95) attached to C-12 in ring D, thus establishing **1** as a new fasamycin congener, for which the name accramycin A is proposed.

Two further accramycin derivatives, **2** and **3**, were tentatively identified in the molecular network (Figure S10) based on their UV spectra, but could not be isolated due to their minute quantities in the extract. The typical chlorine isotope pattern observed for compound **2** (*m/z* 521.1319 ([M + H]^+^: 523.1282, 3:1, both consistent with the molecular formula C_29_H_25_ClO_7_)) in the MN cluster indicated that it was a monochlorinated derivative of **1** (Figures S11 and S12). It is worth noting that chlorination is a feature which is commonly observed in the fasamycin [19] and naphthacemycin [5] series. Compound **3** (*m/z* 535.1478 ([M + H]^+^: 537.1447, 3:1, both consistent with the molecular formula C_30_H_27_ClO_7_)) (*m/z* 535.1500 ([M + H]^+^, C_30_H_28_ClO_7_^+^)) was identified as a homologue of compound **2** (Figure S13–S14). 

### 2.2. Proposed acc Biosynthetic Gene Cluster and Pathway

Given the structural similarity of accramycin A **1** with fasamycins [19], we predicted that **1** is originated from polyketide Type II synthase. In silico analysis of the annotated MA37 genome using antiSMASH 3.0 [20] revealed three putative type 2 PKS biosynthetic gene clusters. One biosynthetic gene cluster (BGC) is likely to be involved in the biosynthesis of accramycins (*acc*) showing high homology to the reported formicamycin BGC (KX859301) [19], and the other two share very high sequence homology to FD-594 (AB469194.1) [21] or hexaricin (KT713752.1) [22] biosynthesis. A pairwise comparison of the annotated proteins using BLAST [23] between the *acc* cluster with the formicamycin BGC showed high amino acid identity, strongly supporting that the *acc* BGC most likely encodes the biosynthesis of accramycins in MA37 (Table S1, Figure S15). 

**Figure 2:**
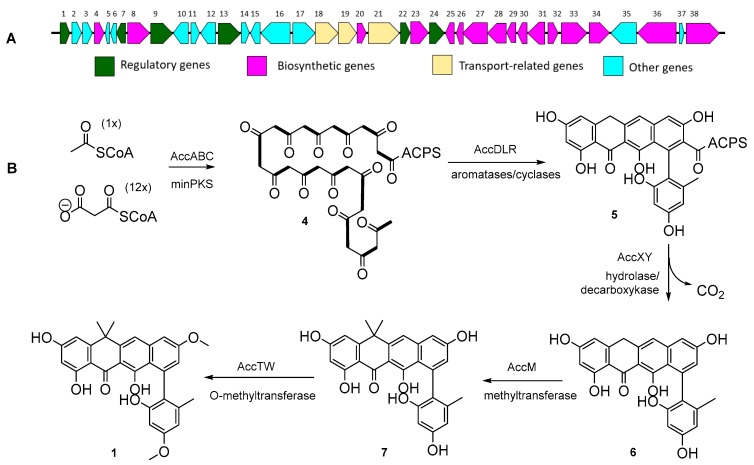
(**A**) Accramycin (*acc*) biosynthetic gene cluster in *Streptomyces sp*. MA37. (**B**) Proposed biosynthetic pathway of **1**.

The *acc* gene cluster spans about 33 kb of genomic DNA and contains sixteen biosynthetic genes with catalytic functions, three putative transport-related genes, and six possible regulatory genes with deduced functions that were designated based on homology analysis (Figure 2A, Table 2). Thirteen open reading frames (*orf* 1–13) in the BGC appear to have no catalytic functions in the biosynthesis of **1**. It is likely that the minimal PKS containing KS_α_ (AccA), KS_β_ (AccB), and ACP (AccC) catalyze the decarboxylative condensation of one acetyl-CoA and twelve malonyl-CoAs to generate a tridecaketide intermediate **4** (Figure 2B), followed by cyclization catalyzed by three cylase/aromatases (AccD, AccL, and AccR) to yield **5**. Further tailoring reactions occur through the action of additional putative enzymes, including PKS hydrolase (AccX) and decarboxylase (AccY) to transform **5** to **6**.

Accramycin A **1** bears two methyl groups at position C-17 in the structure. This *gem*-dimethyl moiety can be found in benastatins [24], resistomysin [25], napthacemycins [5], fasamycins [4], tetarimycin [26], and L-755,805 [27]. Three putative methyltransferases (AccM, AccT, AccW) were identified in the *acc* gene cluster. AccM is closely related to BenF (42%/53%; CAM58795.1) [24,28] which catalyzes the geminal *bis*-methylation step in benastatin biosynthesis, and is likely to involve a similar PKS tailoring reaction in accramycin biosynthesis. The other two methyltransferases, AccT and AccW, showed the highest homology to ForT and ForW (76%/83%, WP_098245764.1; 50%/60%, WP_098245767.1) in the formicamycin pathway, respectively, which are likely to encode the *O*-methylation step of **1–3** at C-12 and C-24.

A further post-PKS transformation involved the halogenation of **1** to produce the minor metabolites **2** and **3**. Analysis of the MA37 genome identified the putative FAD-dependent oxidoreductase AccV, which shows high homology (68%/78%, WP_098245766.1) to ForV halogenase in formicamycin BGC [19] Figure S14, Table S1), and is likely to catalyze the halogenation of **1**, yielding **2** and **3**. 

### 2.3. Antibacterial Assay

Accramycin A **1** showed no activity against Gram-negative bacteria (Table 3). However, it showed moderate activity (MIC 27 µg/mL) against Group B *Streptococcus* (GBS; *Streptococcus agalactiae*) (Figure S16), and this activity has never been reported in any naphthacene-based antibiotics. 

GBS is the leading cause of life-threatening neonatal infections since 1970, and it also affects pregnant women, the elderly, the immunocompromised, and those with underlying chronic medical conditions [29,30]. The case fatality rate among adults has been reported to be markedly higher than among neonates, and continues to climb over the last two decades [29,30,31,32,33]. 

GBS remains susceptible to penicillin; however, increasing MICs (4-fold to 8-fold higher) are now described [33,34]. Reports have shown rising GBS resistance toward erythromycin and clindamycin, ranging between 25–52% and 12–41%, respectively [29,33,34,35,36]. The first two cases of invasive vancomycin-resistant GBS infection were documented in 2014 [37]. Continued investigation is necessary to monitor this emerging threat. The growing incidence of antibiotic resistance reinforces the need to develop alternative drugs.

The current study opens an avenue for possible structural modifications of the accramycin pharmacophore for the development of potent drugs against GBS. Structure–activity relationship studies on the accramycin scaffold would be of interest in the search for potential effective clinical candidates. 

## 3. Materials and Methods

### 3.1. Fermentation

*Streptomyces sp.* MA37 was isolated from the rhizosphere of a Moracear Bark Cloth tree (*Antiaris toxicaria*), growing in the Legon Botanical Gardens, Accra, Ghana, Africa [13]. The seed culture of the MA37 strain was prepared by inoculating a single colony of the organism to 50 mL of ISP2 liquid medium (Oxoid glucose 2.0 g, Oxoid yeast extract 2.0 g, VWR malt extract 5.0 g in 500 mL milliQ H_2_O), and incubating for 7 days at 28 °C with shaking at 180 rpm (Incu-shake FL16-2). The seed culture was used to inoculate 12.0 L of ISP2 broth (1:100) in 24 baffled flasks (Corning^TM^ polycarbonate flasks), each containing already autoclaved 500 mL of ISP2, and plugged with Fisherbrand™ polyurethane foam stoppers (Fisher Scientific, UK). The cultures were incubated for 7 days maintained at 28 °C with continuous agitation at 180 rpm. Subsequently, Diaion® HP-20 (3 g/50 mL) was added under sterile conditions to the fermentation culture and kept at the same temperature and shaking conditions for 18–24 h. The solution was filtered under vacuum, and the HP-20 resin was rinsed with milliQ water, and extracted thrice with 100% methanol (Fisher Chemical HPLC grade). All the methanol extracts were combined, concentrated under reduced pressure (Buchi Rotavapor R200, UK), and subjected to LC high-resolution electrospray ionization mass spectrometry (LC HRESIMS, UK) analysis.

### 3.2. Extraction and Isolation

The crude methanol extract was fractionated on Strata^®^ C18-E solid phase extraction (SPE) (55 µm, 70 Å, 20 g/60 mL) cartridges, and yielded 6 fractions (S1–6). Fractionation was performed using 10 column volumes of milliQ H_2_O (S1), 25% MeOH (S2), by 50% MeOH (S3), followed by 75% MeOH (S4), and then 100% MeOH (S5). Finally, the SPE column was flushed with 100% MeOH with 0.05% of trifluoroacetic acid (S6) (Acros Organics). All the eluents were concentrated under reduced pressure (Buchi Rotavapor R200). Each fraction was subjected to LC-high-resolution electrospray ionization mass spectrometry (HRESIMS) analysis.

HPLC-UV analysis was carried out in all the fractions (S1–6) to screen and target unique scaffolds using an Agilent HPLC system (UK) (1260 Infinity) equipped with a diode array detector (DAD) (C-18 ACE 10 µM 10 × 250 mm column) with spectral scanning between 200–550 nm. The compounds of interest with the characteristic naphthacene UV maxima (226, 250, 286, 355, and 420 nm) were observed in fraction S3. 

Analysis of the HPLC/HRESIMS data of fraction S3 revealed three resolved peaks with [M + H]^+^ at *m/z* 487.1741, 521.1318, and 535.1500, respectively. Dereplication of these masses was achieved using AntiBase (2012) software, indicating that all three molecules were previously unreported. Thus, fraction S3 was selected for further fractionation by reversed-phase semi-prep (C18 ACE 10 µM 10 × 250 mm column) HPLC (Agilent 1260 Infinity). The purification was carried out using a linear gradient from 20% H_2_O:MeOH:TFA (95:5:0.1) to 100% MeOH for 45 min with a solvent flow rate of 1.5 mL/min, to yield accramycin A **1** (2.0 mg). Two further accramycin derivatives, **2** and **3** were identified in fraction S3; however, they were not subjected to isolation due to their minute quantities in the extract.

Accramycin A **1**: deep yellow powder. UV (CH_3_OH): 226, 250, 286, 355, and 420 nm; IR (neat) ν_max_ 3320 cm^−1^, 2945 cm^−1^, 2832 cm^−1^, 1650 cm^−1^, 1403 cm^−1^, 1111 cm^−1^, 1021 cm^−1^; ^1^H, ^13^C NMR data, see Table 1; HRESIMS (positive mode) *m/z* calculated [M + H]^+^ = 487.1751; observed [M + H]^+^ = 487.1741; ∆ = −2.1 ppm.

### 3.3. MS/MS Molecular Networking

The MS/MS data of the crude extract and fractions (S1–6) were converted from .RAW to .mzXML file format using the ProteoWizard MSConvert software (version 3.0) ([38] for metabolite profiling and the identification of new chemical entities in *Streptomyces sp.* MA37 extracts. The .mzXML files were uploaded on the GNPS web platform to create the molecular network [15,39,40]. The GNPS settings used to obtain the consensus spectra were: Precursor ion mass tolerance of 0.02 Da and fragment ion tolerance of 0.02 Da. Edges were filtered by setting the default GNPS minimum cosine score above 0.7, minimum cluster size 2, network TopK 10, and more than 6 matched peaks. The spectral library matching was performed with a similar cosine threshold and minimum matched peaks. The remaining parameters were set as default, as suggested by the GNPS platform. The molecular network was generated using the GNPS “Classic” molecular networking workflow (METABOLOMICS-SNETS-V2 version 1.2.5,), and the GraphML data obtained were visualized using the circular layout in Cytoscape 3.6.1 [41]. 

### 3.4. Spectroscopic Analysis

High Resolution Electro-spray Ionization Mass Spectrometry (HR-ESIMS) was obtained using a LC MS Thermo Scientific MS system (LTQ Orbitrap) coupled to a Thermo Instrument HPLC system (Accela PDA detector, Accela PDA autosampler and Accela Pump) on a positive ESI mode (30.000), MS/MS resolution 7500, C18 (Sunfire 150 × 46 mm column) Reversed phase separation using 0.1% formic acid in water and 0.1% formic acid in acetonitrile gradient. The following conditions were used: Capillary voltage 45 V, auxiliary gas flow rate 10–20 arbitrary units, capillary temperature 200 °C, sheath gas flow rate 5 arbitrary units, spray voltage 4.5 kV, mass range 150–2000 amu (maximum resolution 30,000×). 1D and 2D NMR data were obtained on a Bruker AVANCE III HD 600 MHz (Ascend^TM^14.1 Tesla, UK) with Prodigy TCI^TM^ cryoprobe at 298 K in DMSO-*d_6_* and CD_3_OD. Trimethylsilane (TMS) was used as an internal standard. Deuterated solvents were obtained from Goss Scientific. The infrared spectrum was acquired using a PerkinElmer Spectrum version 10.4.00 Fourier transform infrared (FTIR) spectrometer (2013) (UK) equipped with an Attenuated Total Reflection (ATR) diamond cell. 

### 3.5. Antibacterial Assay

Minimum inhibitory concentrations (MIC) were determined following the antibacterial assay protocols described in Lauritano (2016) [42] against the Gram-negative bacteria *Escherichia coli* (ATCC 25922), *Pseudomonas aeruginosa* (ATCC 27853), and the Gram-positive bacterium *Streptococcus B* (ATCC 12386). All bacteria were cultured in Mueller–Hinton broth. The assays were performed in serial dilutions (in triplicates) in 96-well plates (Nunc, Thermo Fisher Scientific, United States), wherein a 50-µL suspension (log phase) of bacteria was incubated overnight at 37 °C and then supplemented with 50 µL of the test extract. The positive control consisted of the bacteria plus Milli-Q water (no test compound), while the negative control comprised the growth media and Milli-Q water. The absorbance was recorded after 24 h (OD_600_) in a Victor3 multilabel plate reader. The growth medium appeared clear in wells where the test compound prevented the growth or killed the bacteria; otherwise, it was cloudy. Activity threshold was set below 0.05 (OD_600_). The MIC was defined as the lowest concentration of a drug that inhibited visible bacterial growth.

## 4. Conclusions

With the rising GBS incidence and antibiotic resistance, we have yet to find more base scaffolds for the development of alternate drugs and drug leads. Herein, we report the isolation of Accramycin A **1** from the soil bacterium *Streptomyces sp.* MA37 by HPLC-UV aided by molecular networking. The structure of **1** was deduced by analysis of the LC MS, UV, 1D, and 2D NMR, and identified as a new naphthacene-type natural product. Preliminary studies on the antibacterial properties of **1** using GBS showed an MIC of 27 µg/mL. This is the first report of GBS activity amongst the naphthacene-based congeners, and suggests possible scaffold manipulation for the development of potent drugs against GBS based on the accramycin framework. Two accramycin analogues, **2** and **3**, were tentatively identified in the MA37 fermentation broth by HPLC/HRESIMS analysis and molecular network. A putative accramycin biosynthetic pathway was proposed.

## Figures and Tables

**Table 1:** NMR data for accramycin A **1** (CD_3_OD, 600 MHz at 298 K).

Position	^13^C ^a^	^1^H, mult. (*J*, Hz)	Position	^13^C ^a^	^1^H, mult. (*J*, Hz)
1	112.1, CH	6.49, d (2.4)	16	147.4, C	-
2	167.0, C ^b^	-	17	39.3, C	-
3	104.0, CH	5.99, d (2.4)	18	154.5, C	-
4	167.1, C	-	19	34.4, CH_3_	1.70, s
5	105.0, C	-	20	34.7, CH_3_	1.69, s
6	189.2, C	-	21	125.3, C	-
7	107.4, C	-	22	155.2, C	-
8	165.3, C	-	23	99.2, CH	6.32, d (2.4)
9	118.7, C	-	24	160.2, C	-
10	141.0, C ^b^	-	25	106.9, CH	6.37, d (2.4)
11	121.7, CH	6.71, d (2.4)	26	138.2, C	-
12	161.1, C	-	27	20.6, CH_3_	1.91, s
13	106.6, CH	7.19, d (2.4)	28	55.3, CH_3_	3.80, s
14	141.8, C	-	29	55.6, CH_3_	3.95, s
15	115.8, CH	7.47, s			

^a^ Obtained from HSQC and HMBC, respectively; ^b^ estimated from DMSO-*d_6_* spectrum.

**Table 2:** Deduced functions of open reading frames (ORFs) in accramycin (*acc*) biosynthetic gene cluster.

No.	Gene	AA	Deduced Function	No.	Gene	AA	Deduced Function
1	*accE*	242	DNA binding response regulator	20	*accL*	119	Type II PKS cyclase
2	*orf1*	226	Synthase	21	*accK*	509	Na^+^/H^+^ exchanger
3	*orf2*	210	Glycosyl transferase	22	*accJ*	145	MarR family transcriptional regulator
4	*accM*	220	SAM-dependent methyl transferase	23	*accG*	359	Sensor histidine kinase
5	*orf3*	60	Hypothetical protein	24	*accF*	227	LuxR family response regulator
6	*orf4*	38	Unknown	25	*accD*	152	Polyketide cyclase
7	*accN*	112	Transcriptional regulator	26	*accC*	97	Acyl carrier protein
8	*accO*	305	NAD(P)-dependent oxidoreductase	27	*accB*	415	Beta-ketoacyl synthase
9	*accP*	275	MerR family transcriptional regulator	28	*accA*	426	Beta-ketoacyl synthase
10	*orf5*	193	Nucleoside phosphorylase	29	*accR*	131	Cupin (cyclase/monooxygenase)
11	*orf6*	146	VOC family protein	30	*accS*	113	Antibiotic biosynthesis monooxygenase
12	*orf7*	202	Isomerase	31	*accT*	350	*O*-methyl transferase
13	*accI*	303	LysR family transcriptional regulator	32	*accU*	113	Antibiotic biosynthesis monooxygenase
14	*orf8*	108	Hypothetical protein	33	*accV*	430	Halogenase
15	*orf9*	140	Hypothetical protein	34	*accW*	348	*O*-methyl transferase
16	*orf10*	386	Molybdopterin-binding protein	35	*orf12*	480	Phenylalanine specific permease
17	*orf11*	480	domain containing proteins	36	*accX*	777	hydrolase
18	*accH*	491	ABC transporter	37	*orf13*	43	DUF-1232 domain containing protein
19	*accI*	319	ABC transporter	38	*accY*	517	decarboxylase

**Table 3:** Minimum inhibitory concentration (MIC) of accramycin A **1** against a panel of bacterial pathogens.

Pathogen	MIC (µg/mL)
*Streptococcus B.* ATCC 12386	27
*Escherichia coli* ATCC 25922	>50
*Pseudomonas aeruginosa* ATCC 27853	>50

## Supplementary Materials

The supplementary materials are available online.
