# Supplementary material for: Accramycin A, A New Aromatic Polyketide, from the Soil Bacterium, Streptomyces sp. MA37
Source: Molecules. 2019 Sep 17;24(18):3384. doi: 10.3390/molecules24183384 (PMC6767120; doi:10.3390/molecules24183384)
Supplement: Supplementary file 1 [file molecules-24-03384-s001.pdf]

SUPPLEMENTARY INFORMATION

Accramycin A, a new aromatic polyketide, from the soil bacterium, *Streptomyces sp.* MA37

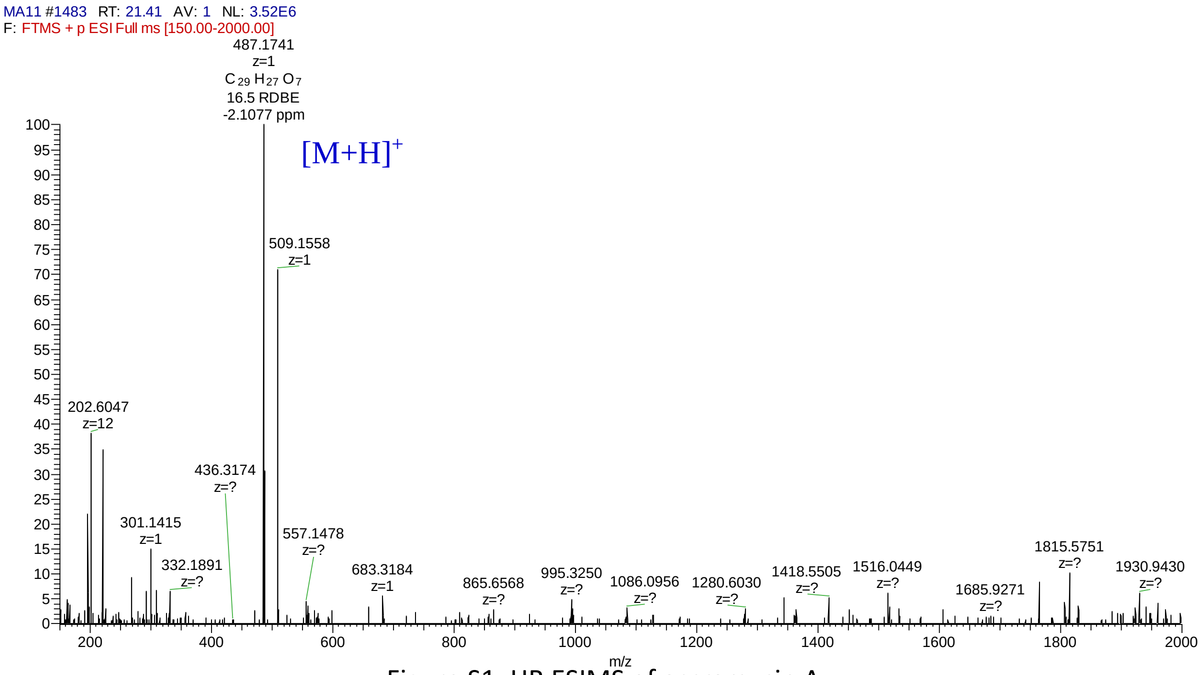

Figure S1. HR ESIMS of accramycin A

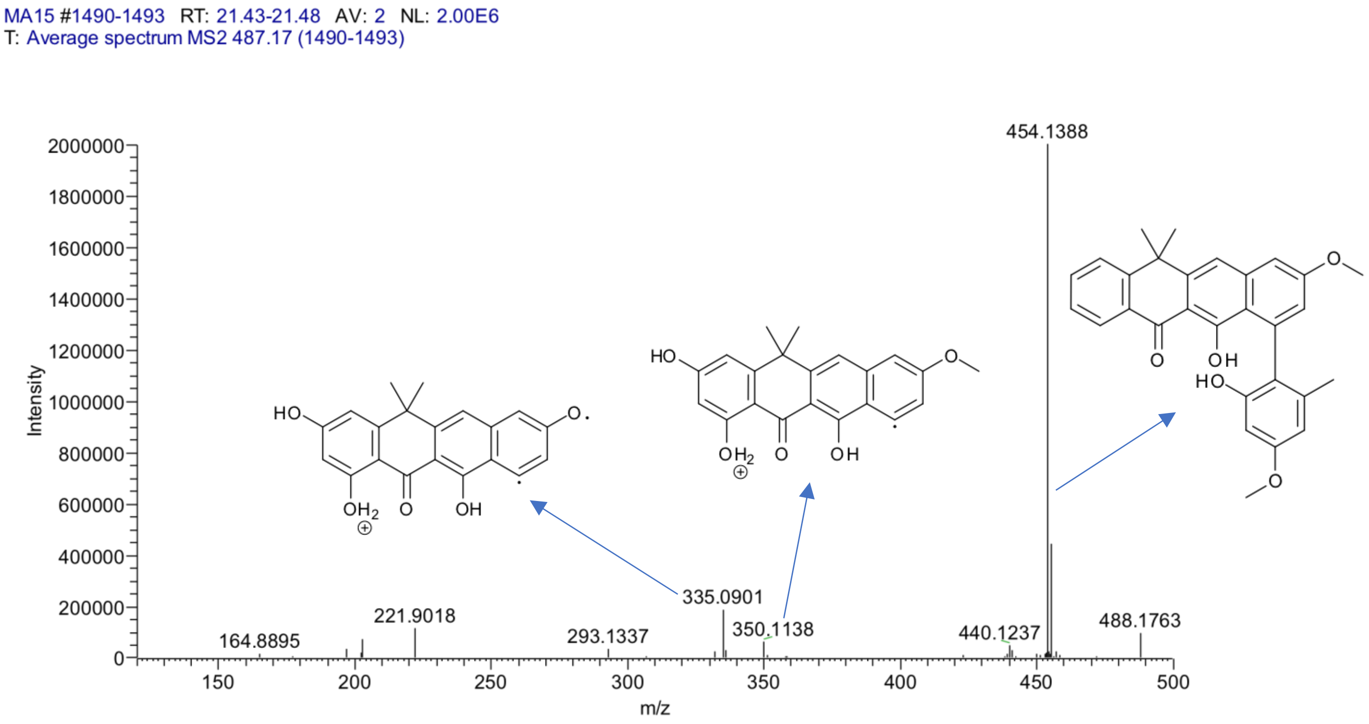

Figure S2. MS/MS fragmentation of accramycin A

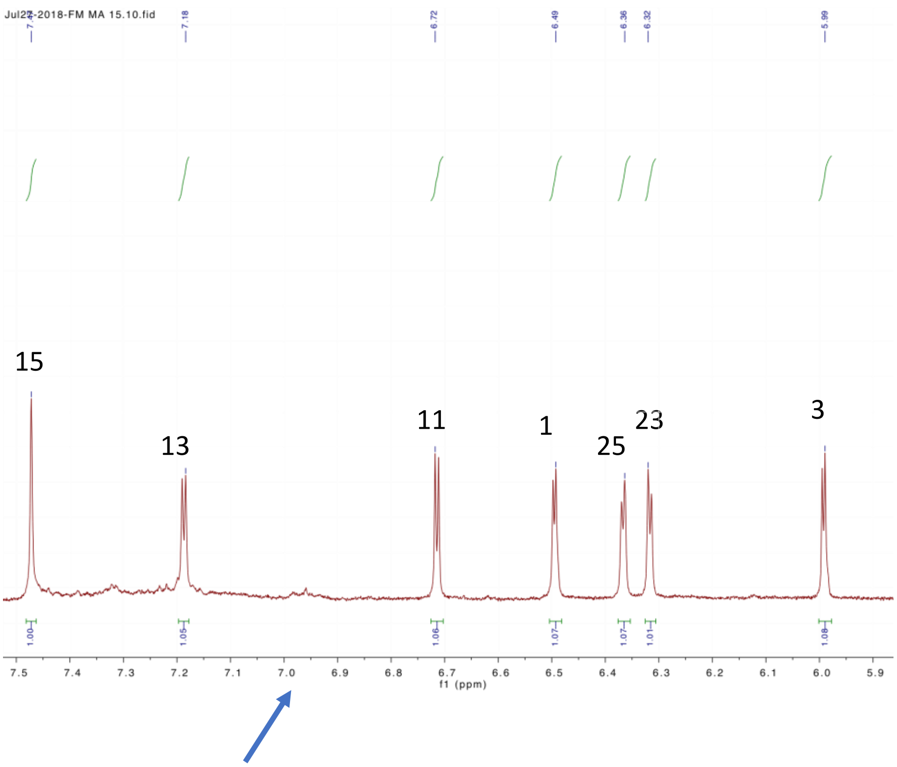

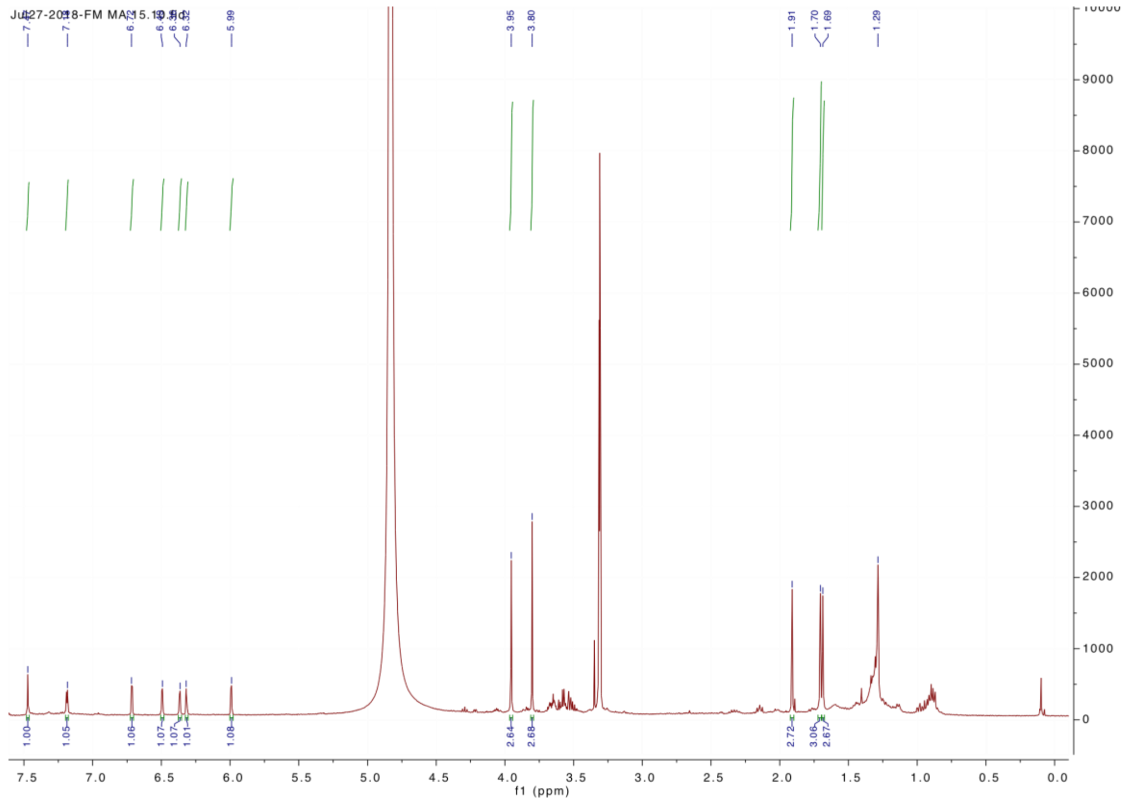

Figure S3. ^{1}H-NMR spectrum of accramycin A in CD_3OD at 600 MHz

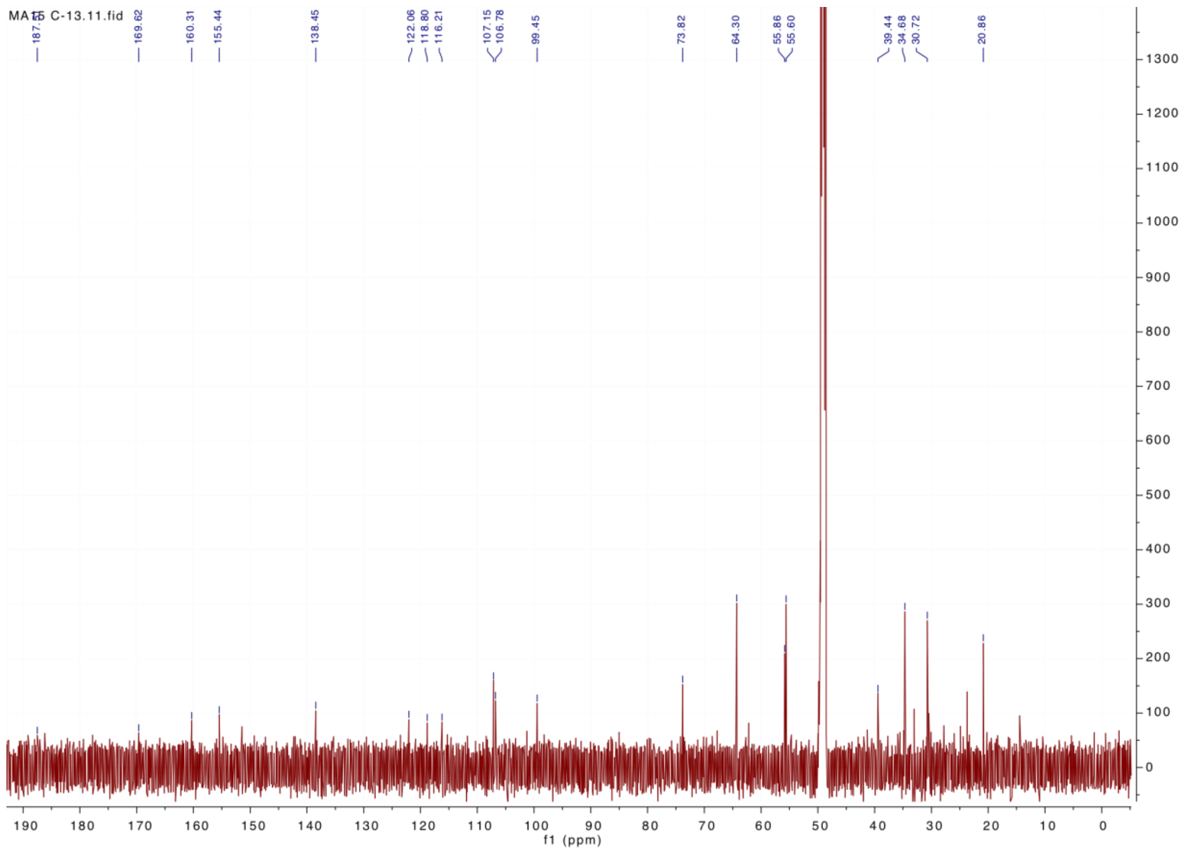

Figure S4. ^{13}C NMR spectrum of accramycin A in CD_3OD at 600 MHz

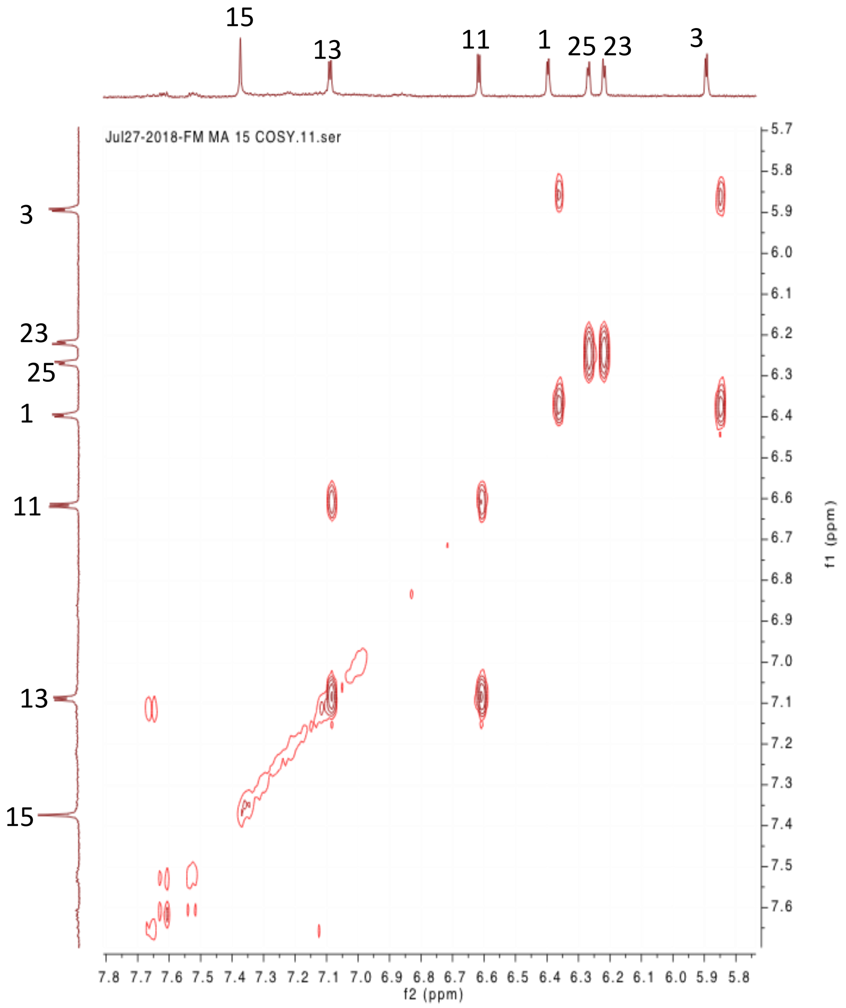

Figure S5. COSY spectrum of accramycin A in CD_3OD at 600 MHz

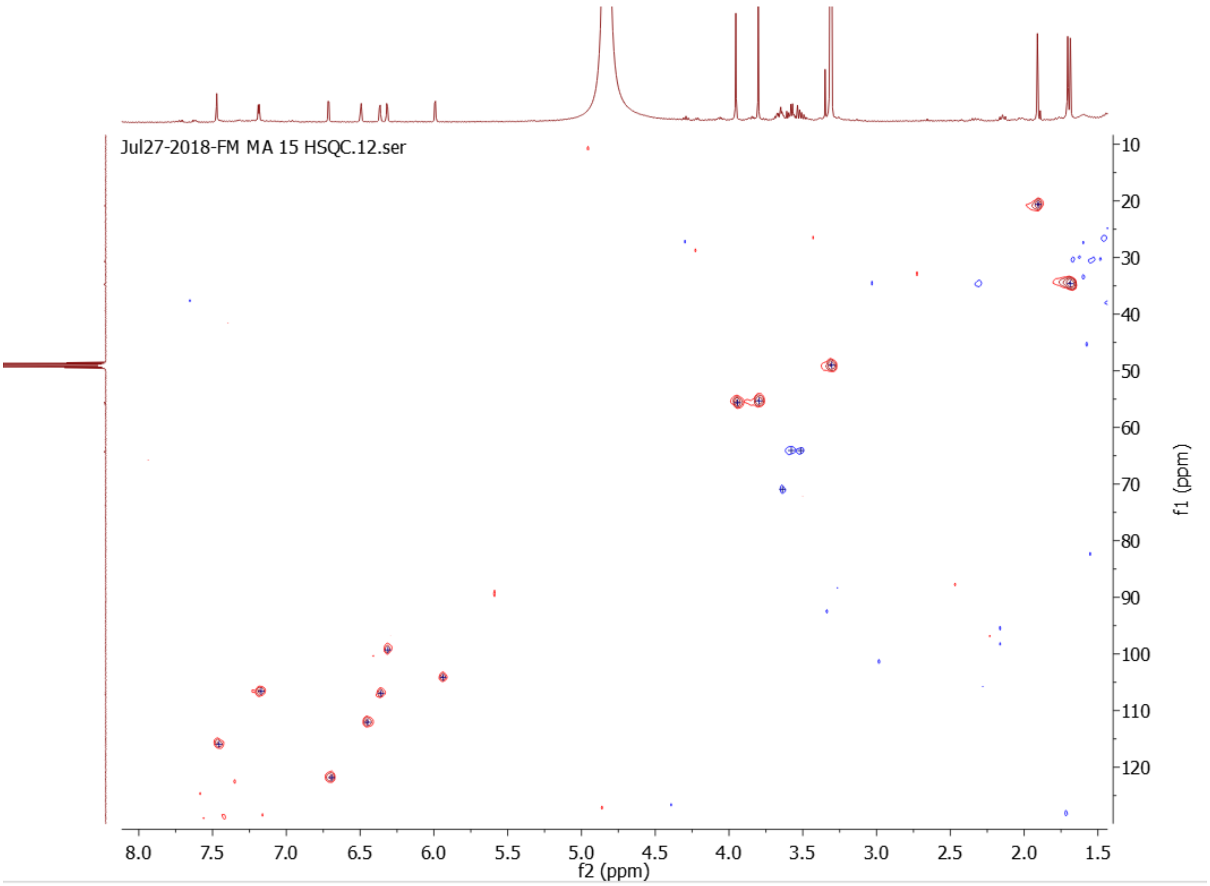

Figure S6. HSQC spectrum of accramycin A in CD_3OD at 600 MHz

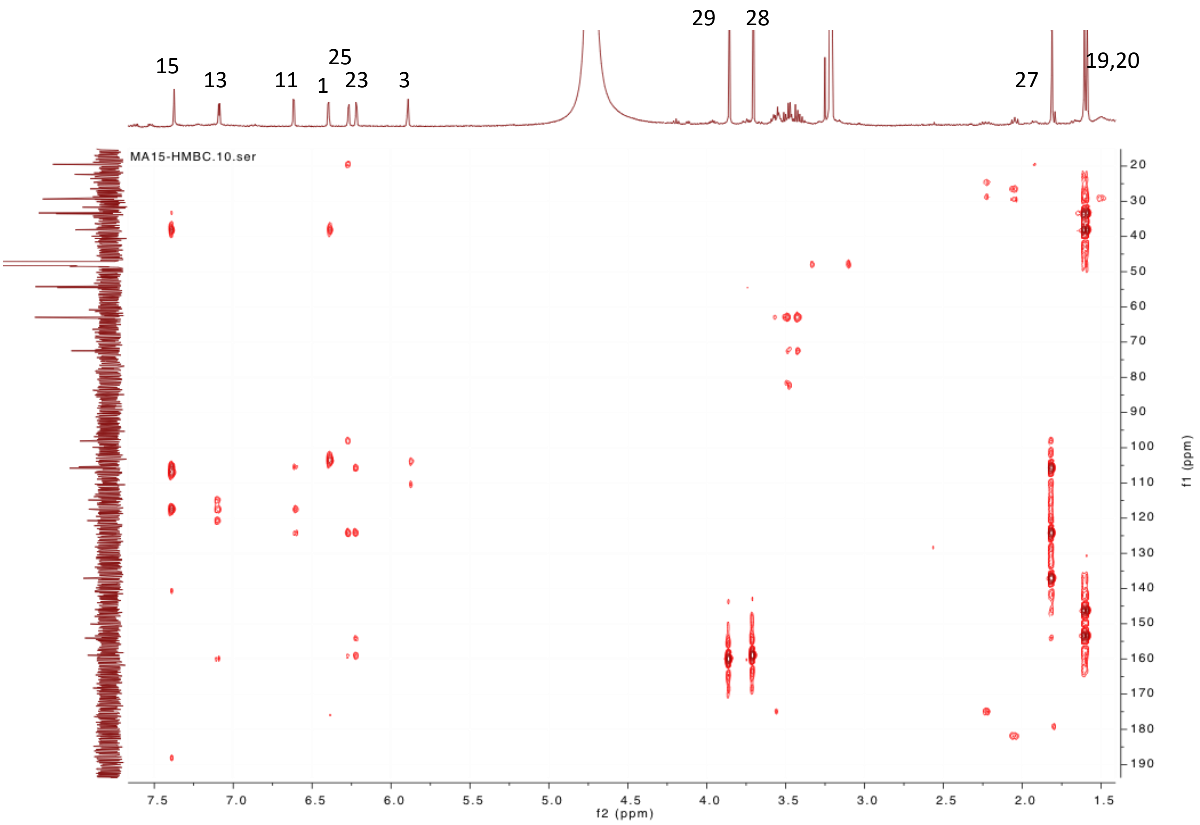

Figure S7. HMBC spectrum of accramycin A in CD_3OD at 600 MHz

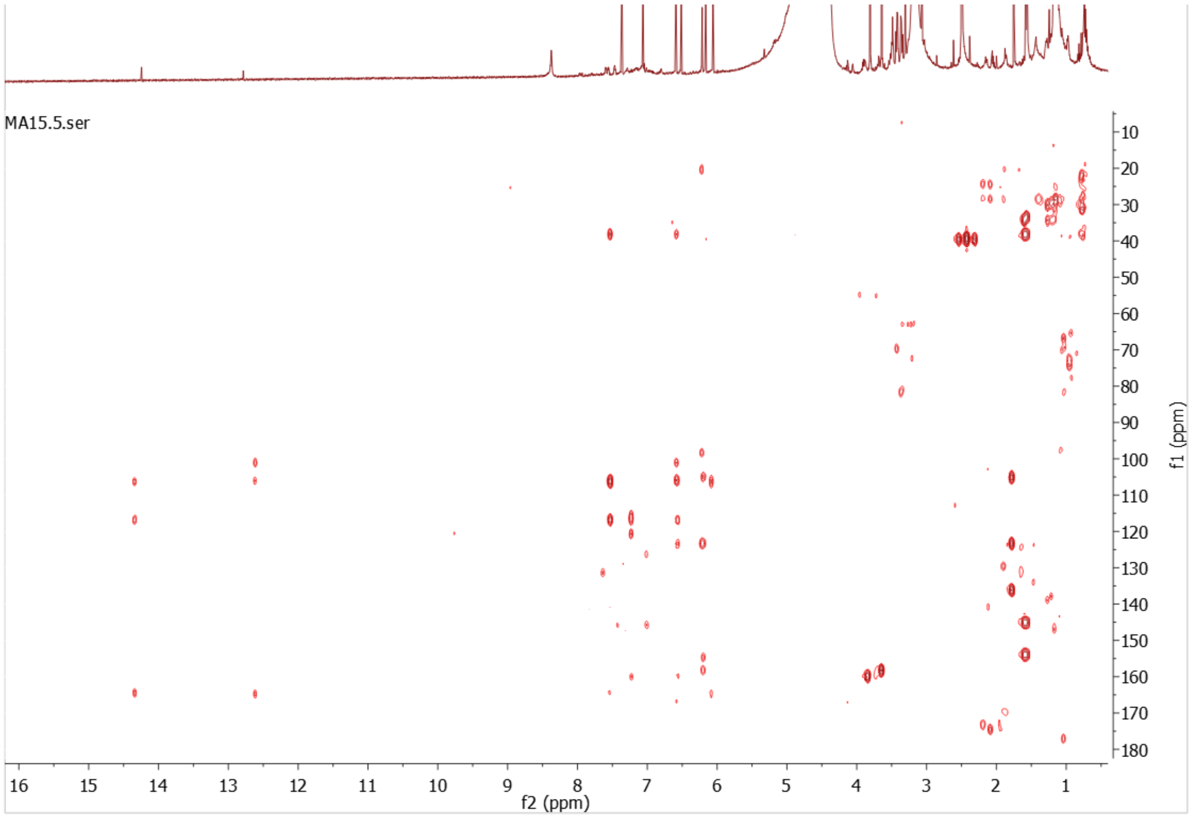

Figure S8. HMBC spectrum of accramycin A in DMSO-d_6 at 600 MHz

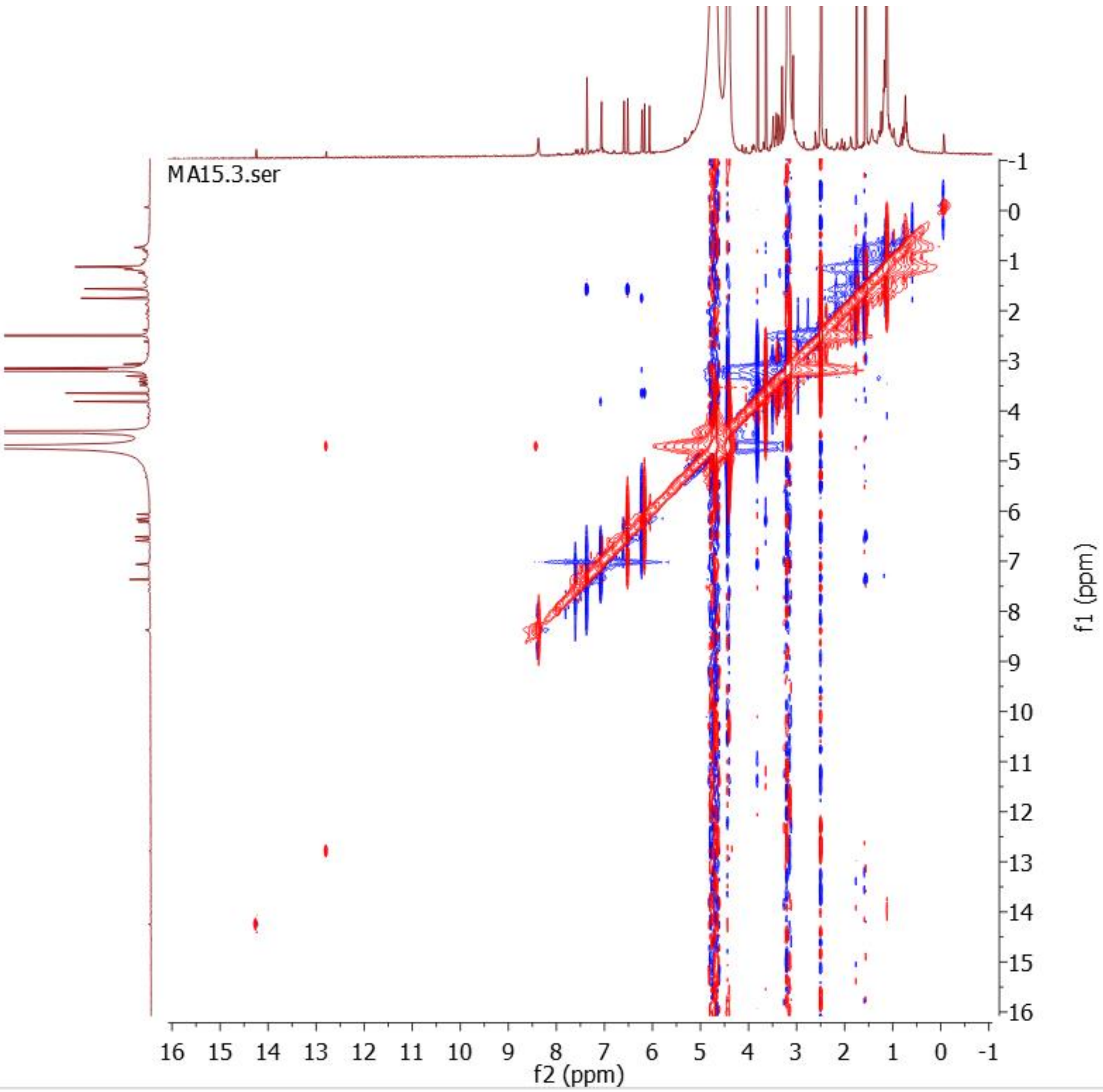

Figure S9. NOESY spectrum of accramycin A in DMSO-d_6 at 600 MHz

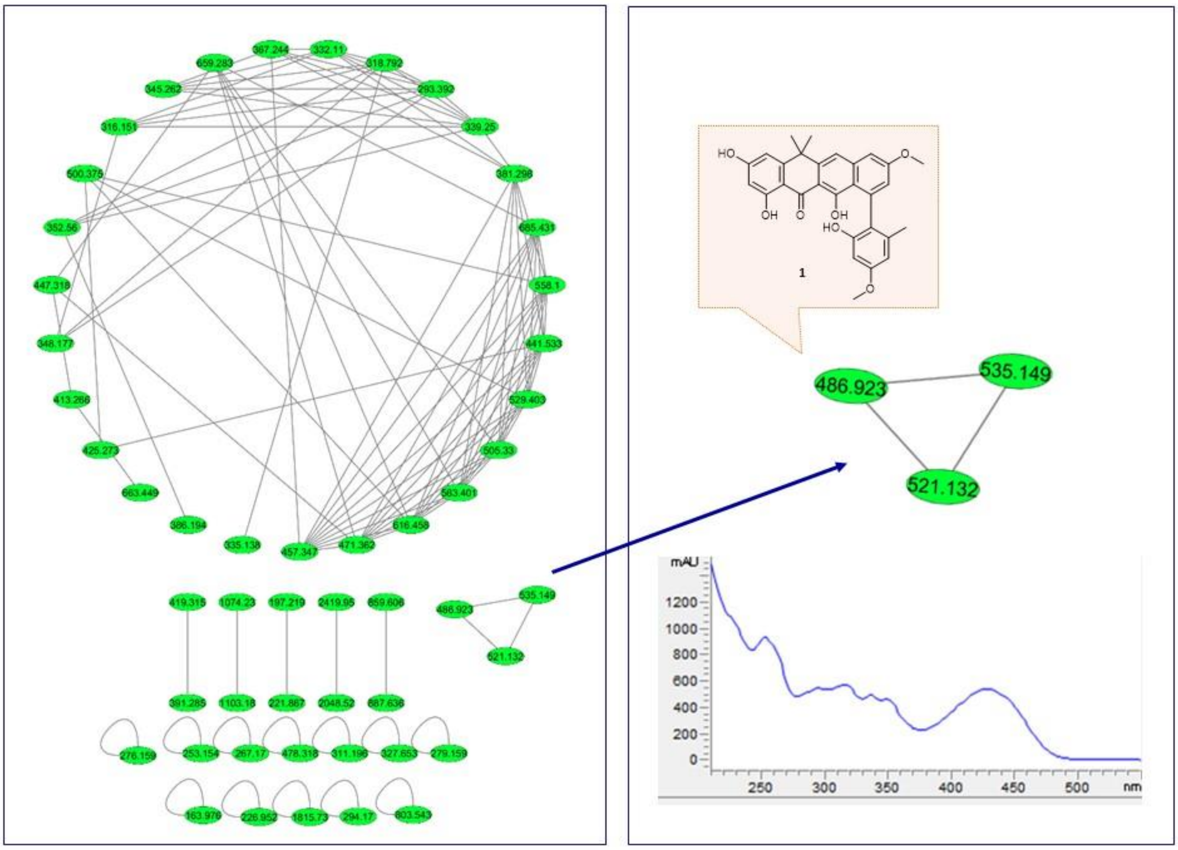

Figure S10. Accramycin cluster from the molecular network of MA37 extract and the corresponding UV spectra (200-550 nm)

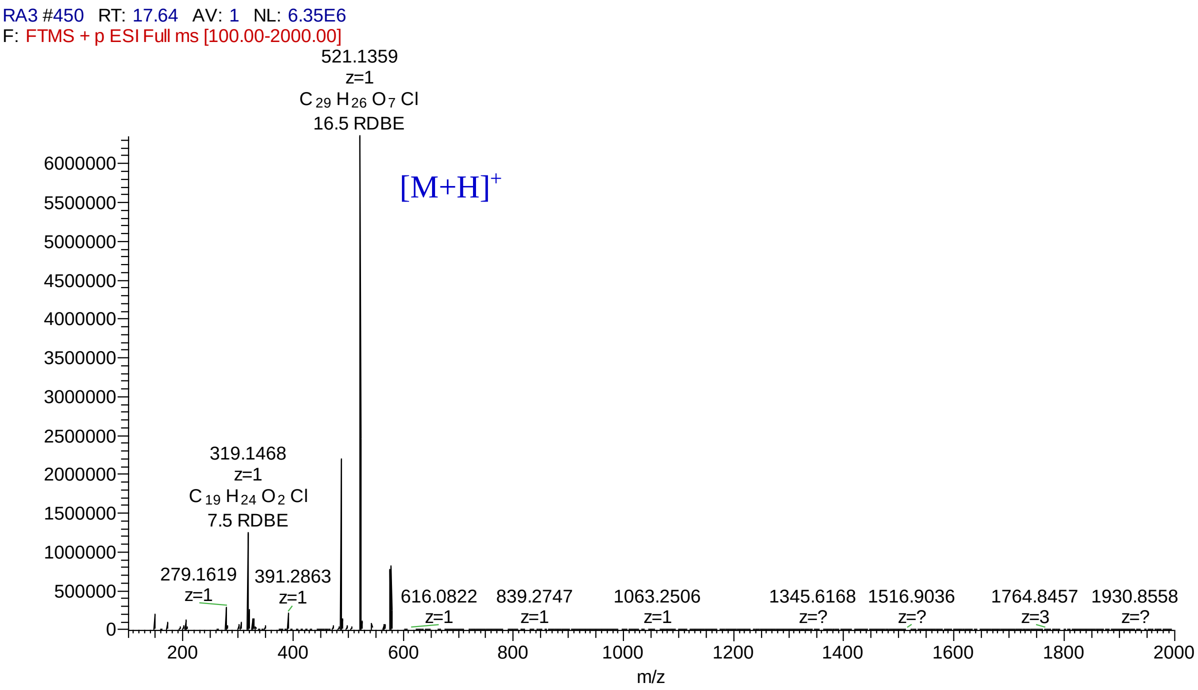

Figure S11. HR ESIMS of accramycin derivative **2**

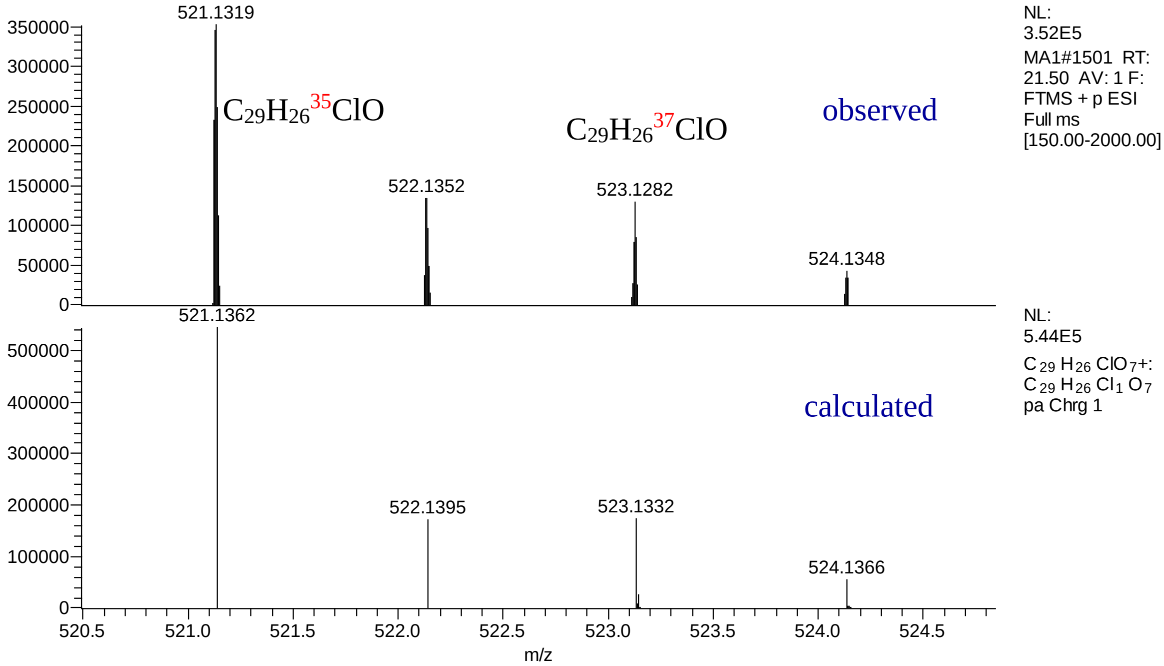

Figure S12. Isotope pattern of accramycin derivative **2**

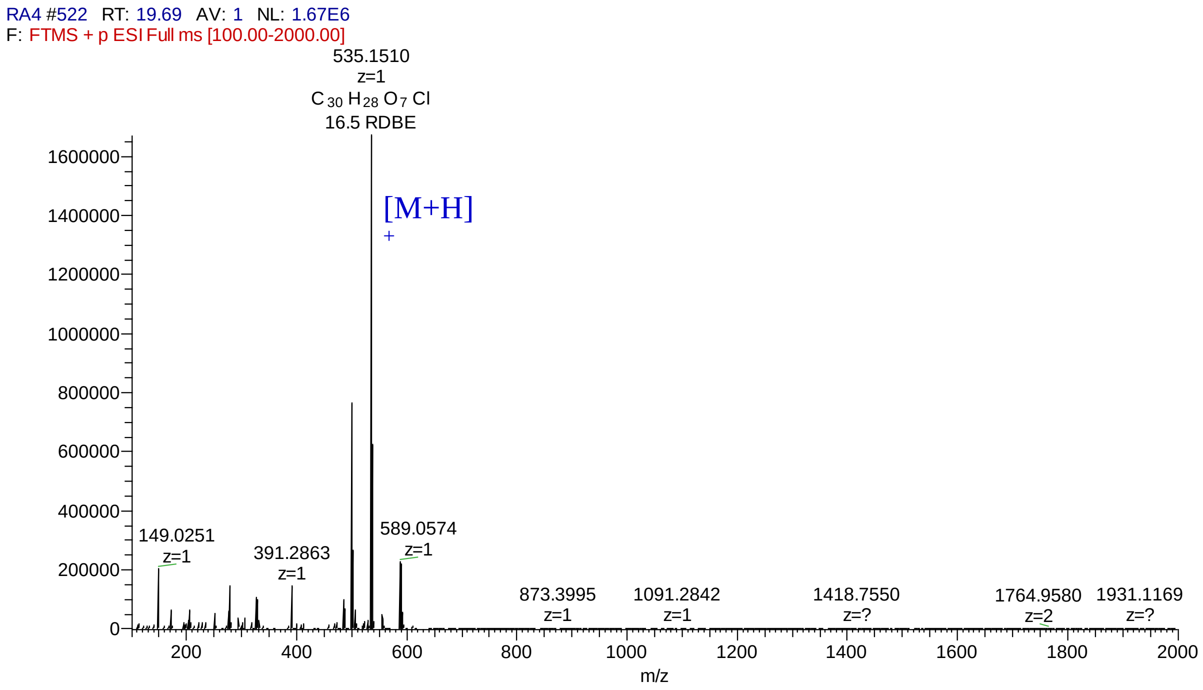

Figure S13. HR ESIMS of accramycin derivative **3**

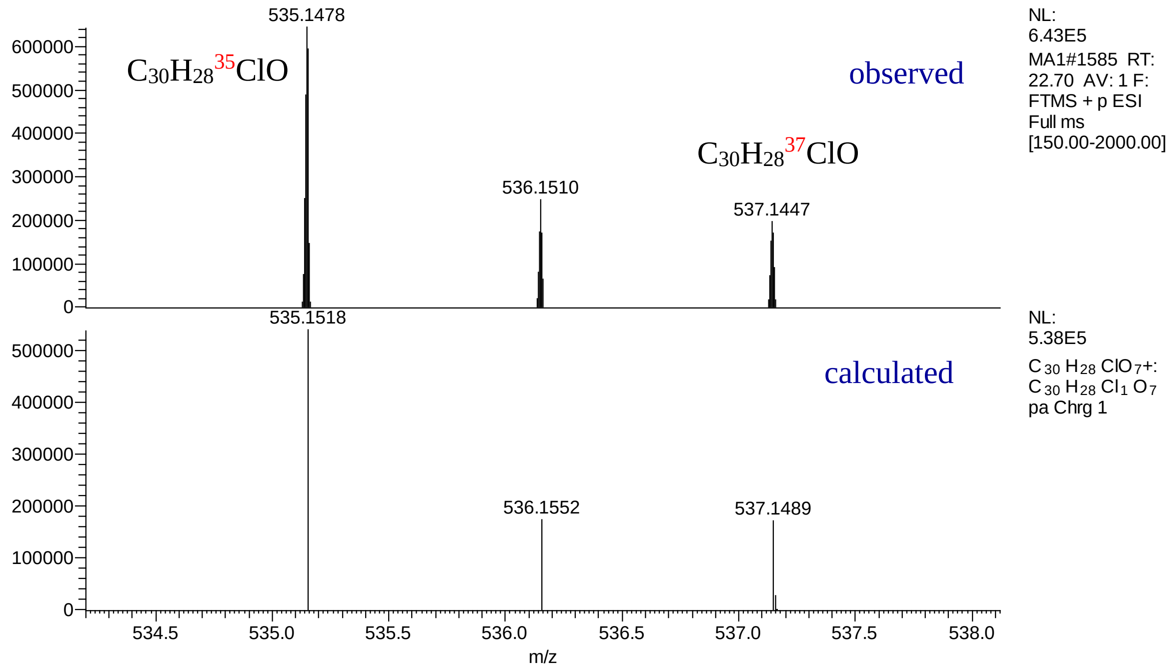

Figure S14. Isotope pattern of accramycin derivative **3**

Table S1. Deduced functions of the ORFs in the *acc* biosynthetic gene cluster in *Streptomyces* sp. MA37. Highlighted in blue those that showed closest homology to the formicamycin (*for*) BGC in *Streptomyces formicae*.

	Gene	AA	Deduced Function	Closest similarity in database	Identities / Positives
1	*accE*	242	DNA binding response regulator	*Streptomyces albireticuli*	88% / 93%
2	*orf1*	226	Synthase	*Streptomyces orinoci*	85% / 88%
3	*orf2*	210	Glycosyl transferase	*Streptomyces sp. CB01635*	74 % / 78%
4	*accM*	220	SAM-dependent methyl transferase	*Streptomyces sp. TLI_146*	75% / 81%
5	*orf3*	60	Hypothetical protein	*Streptomyces glaucescens*	71% / 80%
6	*orf4*	38	Unknown		
7	*accN*	112	Transcriptional regulator	*Streptomyces antibioticus*	93 % / 95 %
8	*accO*	305	NAD(P) dependent oxidoreductase	*Streptomyces sp. MUSC 93*	87% / 92%
9	*accP*	275	MerR family transcriptional regulator	*Kitasatospora mediocidica*	76% / 86%
10	*orf5*	193	Nucleoside phosphorylase	*Saccharothrix sp. ST-888*	68% / 83%
11	*orf6*	146	VOC family protein	*Streptomyces orinoci*	82% / 88%
12	*orf7*	202	Isomerase	*Streptomyces globisporus]*	68% / 78%
13	*accI*	303	LysR family transcriptional regulator	*Streptomyces sp. NEAU-D10*	75% / 82%
14	*orf8*	108	Hypothetical protein	*Streptomyces sp. TLI_146*	79% / 87%
15	*orf9*	140	Hypothetical protein	*Streptomyces roseoverticillatus*	60% / 65%
16	*orf10*	386	Molybdopterin-binding protein	*Streptomyces olivoreticuli*	82% / 88%
17	*orf11*	480	Domain containing proteins	*Streptomyces olivoreticuli*	76% / 81%
18	*accH*	491	ABC transporter	*Streptomyces sp. MBT76*	88% / 92%
19	*accI*	319	ABC transporter	*Streptomyces cinnamoneus*	86% / 90%
20	*accL*	119	Type II PKS cyclase	*Streptomyces*	73% / 80%
21	*accK*	509	Na+/H+ exchanger	*S. formicae*	69 % / 76%
22	*accJ*	145	MarR family transcriptional regulator	*S. formicae*	68% / 77%
23	*accG*	359	Sensor histidine kinase	*S. formicae*	53% / 67%
24	*accF*	227	LuxR family response regulator	*S. formicae*	74% / 82%
25	*accD*	152	Polyketide cyclase / dehydrase	*Streptomyces cyaneogriseus*	74% / 84%
26	*accC*	97	Acyl carrier protein	*S. formicae*	56% / 66%
27	*accB*	415	Beta-ketoacyl synthase	*S. formicae*	75% / 81%
28	*accA*	426	Beta-ketoacyl synthase	*S. formicae*	79% / 86%
29	*accR*	131	cupin (cyclase / monooxygenase)	*S. formicae*	76% / 82%
30	*accS*	113	Antibiotic biosynthesis monooxygenase	*S. formicae*	66% / 73%
31	*accT*	350	O-methyl transferase	*S. formicae*	76% / 83%
32	*accU*	113	Antibiotic biosynthesis monooxygenase	*S. formicae*	82% / 88%
33	*accV*	430	Halogenase	*S. formicae*	68% / 78%
34	*accW*	348	O-methyl transferase	*S. formicae*	50% / 60%
35	*orf13*	480	Phenylalanine specific permease	*Streptomyces luteoverticillatus*	91% / 94%
36	*accX*	777	Hydrolase	*Streptomyces alboverticillatus*	82% / 90%
37	*orf14*	43	DUF-1232 domain containing protein	*Deinococcus yavapaiensis*	44% / 48%
38	*accY*	517	Decarboxylase	*Streptomyces varsoviensis*	72% / 79%

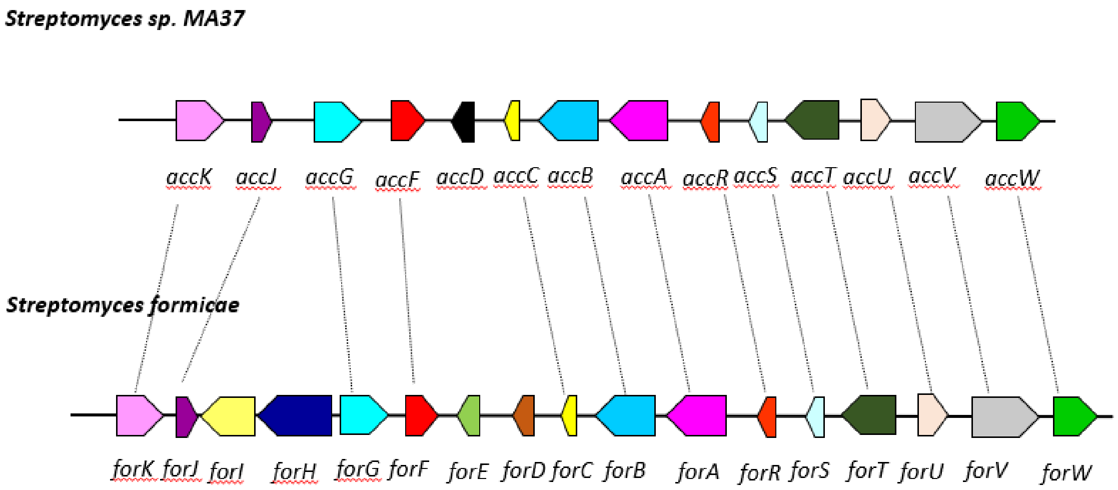

Figure S15. Comparison of the genes surrounding the *accV* halogenase gene of *Streptomyces* sp. MA37 vs. *Streptomyces formicae*

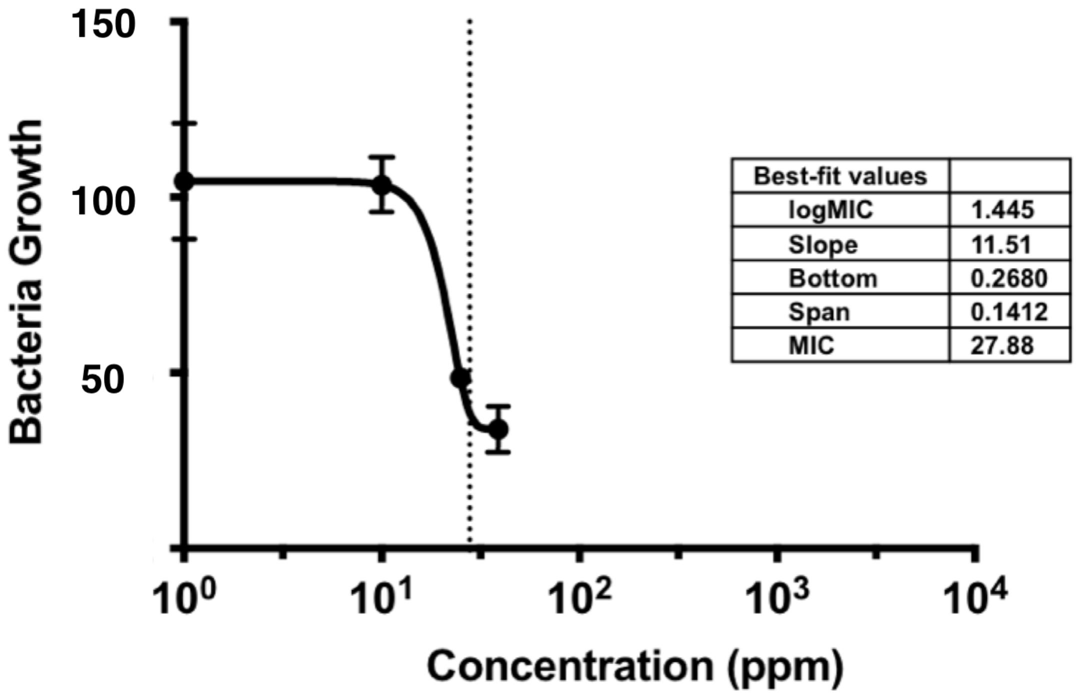

Figure S16. MIC curve of accramycin A against *Streptococcus B.* ATCC 12386 using *Gompertz* model
